# Predicting health outcomes in dogs using insurance claims data

**DOI:** 10.1038/s41598-023-36023-5

**Published:** 2023-06-05

**Authors:** Christian Debes, Johannes Wowra, Sarosh Manzoor, Audrey Ruple

**Affiliations:** 1 Spryfox GmbH, Darmstadt, Germany; 2 Virginia Polytechnic Institute and State University, Blacksburg, USA

**Keywords:** Diseases, Information technology

## Abstract

In this paper we propose a machine learning-based approach to predict a multitude of insurance claim categories related to canine diseases. We introduce several machine learning approaches that are evaluated on a pet insurance dataset consisting of 785,565 dogs from the US and Canada whose insurance claims have been recorded over 17 years. 270,203 dogs with a long insurance tenure were used to train a model while the inference is applicable to all dogs in the dataset. Through this analysis we demonstrate that with this richness of data, supported by the right feature engineering, and machine learning approaches, 45 disease categories can be predicted with high accuracy.

## Introduction

### Topic motivation and significance

The global pet care market was estimated at 179.4 billion dollars in 2020 and expected to grow to 241.1 billion dollars by 2026^1^. The pet insurance market alone is forecasted to grow from 4.5 billion to 16.8 billion by 2030^2^. These numbers reflect not only the growth in pet ownership (currently at 84.5 million households in the US^3^ and 88 million households in Europe^4^), but also the increased needs of pet owners to invest in the health of their companions^5–7^. Data shows that more families decide to adopt pets^8^ and the pet’s role is increasingly changing from being considered property to being regarded as a family member^9–11^. As part of this development, there is a rising need for and willingness to invest in premium healthcare and wellness products.

Pet health prediction aims at understanding the impact of factors surrounding a pet’s life (such as age, breed, pre-existing health conditions, environment, lifestyle, etc.) on its future health. Examples of these factors include:Pre-existing condition-based

- *Pre-existing condition-based*: Dogs diagnosed with developmental disorders have a higher risk of gait abnormalities in the future^12,13^.
- *Breed-based*: English and French bulldogs have a higher risk of dermatological diseases when compared to other breeds of dogs^14,15^.
- *Environment-based (1)*: Dogs living in a warmer climate have a higher risk of developing arthritis^16^.
- *Environment-based (2)*: Dogs living in urban areas have a higher risk of skin diseases^17,18^.
- *Sex-based*: Female dogs have a higher risk of developing urinary tract infections than do male dogs^19,20^.
- *Age-based*: Older dogs have an increased risk of developing cancer than younger dogs^21,22^.

Some of these associations between predictive variables and health outcomes have previously been reported in the veterinary literature. However, risk analyses have historically been conducted in populations of limited individuals, leading to use of small sample sizes, and often investigated only a single health outcome at a time^23–25^. The power of health prediction using machine learning-based approaches, is that data associated with a large number of dogs can be analyzed simultaneously and quantitative results obtained, which link the aforementioned factors. This allows for a much deeper and more refined understanding of the contribution that each factor has upon the future health of dogs.

As such, health prediction is a crucial piece of technology that can be used to help achieve longer and healthier lives for dogs. As a base technology, it allows for multiple analyses, applications, and products to be combined, including:

- *Recommendations*: Predicted disease outcomes can be paired with health and lifestyle recommendations, which enables proactive decision-making that reduces the risk of disease outcomes in the future. These actions include (but are not limited to): changes in nutrition, activity types and level of intensity, alerts to early signs of health diagnoses, addition of appropriate medication or preventives and reminders for medical checkups.
- *Actuarial/Pricing*: An understanding of individual disease likelihood allows for a more discrete and fair approach to pricing of insurance policies.
- Research studies: The impact of other external factors e.g., COVID-19 pandemic, seasonal effects, or the availability of new treatment forms, can be studied in near-real time.

In this paper we describe the methods used to create a machine learning-based approach to pet health prediction and evaluate its performance through a series of experiments. Using 2.4 million insurance claims from 785,565 dogs insured under Fetch, Inc. plans were taken as a proxy to model disease outcomes. The paper is structured as follows: This Section "Introduction" includes the motivation and reviews the state of the art in machine learning approaches for pet health prediction and positions this contribution with respect to existing work. As far as the authors are aware, this rich dataset is applied within a scientific context for the first time. Section "Methods" describes the dataset in detail including individual data regarding health conditions, breed, age, sex, geographic and environmental factors such as average temperature and precipitation values, and human population density of dog’s locality. A set of preprocessing steps are necessary as described in Section "Preprocessing", before the machine learning approaches can be employed, which are covered in Section "Proposed approach". The series of conducted experiments and the detailed results are then presented in Section "Results and discussion". We conclude with a summary and outlook in Section "Conclusion".

### State of the art: machine learning approaches for pet health

In the field of pet health prediction, narrow prediction approaches using physical parameters such as blood samples or radiographic imagery have been used to predict specific disease outcomes. Examples include the prediction of canine chronic kidney disease using blood count and urinalysis^26^, and Cushing’s syndrome prediction in dogs using a variety of laboratory measurements^27^. To date, there is no consistent approach aiming to predict multiple disease categories from pet insurance claims. Previous work with pet insurance datasets have resulted in several publications that report associations between particular dog breeds and health outcomes^28–30^ and age-related changes in dog populations^31–33^. However, to the authors’ knowledge, predictive models for multiple disease outcomes in a diverse population of dogs constructed with insurance data have not been previously reported.

## Methods

### Dataset description

The data used in this project includes breed, age, sex and home location information for 785,565 dogs together with diagnosed diseases and treatments employed. These data were collected over 17 years by the pet insurance company Fetch, Inc. and has been fully de-identified. The unique IDs assigned to dogs are generated through a hash function and no personally identifiable information on the pet owners is included in the dataset.

#### Breeds

The original dataset contains more than 500 individual breeds which were grouped into 20 breed groups as described in this section. Some of the individual breeds contained in the dataset, such as Golden retrievers, are well represented in terms of proportion of the total population size, but many of the breeds are less numerous in overall population size. This can become problematic in terms of having a small sample size because it can introduce large variances in the results, especially when moving towards a statistical or machine learning approach in which stratification based on a combination of breed, age, sex, etc., is performed. Thus, individual breeds with small sample sizes were combined using the results of allele-sharing phylograms, haplotype-sharing cladograms, and neighbor-joining trees representing the genetic relationships between various breeds^34,35^. Table 1 shows the distribution of these 20 breed groups. Subgroups for some of these breed categories were created using specific features of breeds contained in the primary breed groups, for instance the ”Terriers” breed group is subdivided into ”Small Terriers” and ”Large Terriers.” Individual breeds combined within each breed grouping as well as subgroupings can be found in Supplemental Table S1.

For this work, mixed breed dogs were classified in one of three ways: (1) when the breeds of both parents were known, the cross breed was included as a first filial generation of hybrid (e.g. when the dog was reported as Labrador retriever crossed with a Poodle, the breed was reported as Labradoodle); (2) when the breed of only one parent was known, the dog was listed as a cross of the predominant breed (e.g. when the dog was reported as Labrador retriever cross, the breed was reported as Labrador mix); (3) when no information was available about the lineage of a mixed breed dog, its breed was reported as mixed and then differentiated by size categories (up to 22.9 pounds = small, 23–70.9 pounds = medium, or 71 pounds or more = large).

**Table 1:** Distribution of breed groups.

Breed group	Population (percentage)
Mixed medium	130,677 (16.63%)
Mixed small	91,803 (11.69%)
Mastiff-like group 1	70,171 (8.93%)
Toy—other	69,761 (8.88%)
Terriers	57,469 (7.32%)
Labs	39,327 (5.01%)
Mixed other	34,192 (4.35%)
Chihuahua	32,811 (4.18%)
Ancient and Spitz	31,837 (4.05%)
Australian-like	31,684 (4.03%)
Shepherd	31,212 (3.97%)
Spaniels	28,714 (3.66%)
Mixed lab and golden	25,535 (3.25%)
Mixed large	23,335 (2.97%)
Golden	23,234 (2.96%)
Dachshund	15,684 (2.00%)
Working dogs—Non-sport	14,042 (1.79%)
Hound	13,343 (1.7%)
Herding dogs—other	11,975 (1.52%)
Mastiff-like group 2	8759 (1.11%)

#### Conditions

Table 2 provides the 20 (out of 1043) most common health conditions that are available in the data set. Similar to the breed distribution, we observed that a large number of conditions requires aggregation so that after stratification with respect to breed, age, sex, etc., a significant number of samples are available per dependent variable. This problem will be addressed via a disease grouping strategy in Section "Preprocessing".

**Table 2:** Distribution of conditions.

Conditions	Number of claims (percentage)
Unspecified allergies	100,458 (4.09%)
Routine treatment	91,138 (3.71%)
Lameness	79,624 (3.24%)
Cruciate ligament tear/rupture	66,834 (2.72%)
Atopy/atopic dermatitis	62,894 (2.56%)
Mass	61,827 (2.52%)
Diarrhea	54,154 (2.21%)
Otitis externa	51,699 (2.11%)
Seizures	47,747 (1.94%)
Urinary tract infection	46,542 (1.90%)
Arthritis/DJD	43,838 (1.79%)
Hepatopathy	43,283 (1.76%)
Gastrointestinal/digestive system disorder	39,575 (1.61%)
ear infection	37,269 (1.52%)
Periodontal disease	37,044 (1.51%)
Vomiting	36,557 (1.49%)
Vomiting and diarrhea	33,363 (1.36%)
Diabetes mellitus	32,479 (1.32%)
Heart murmur	29,031 (1.18%)
Back pain	28,822 (1.17%)
Other	14,31,664 (58.30%)

#### Age

The dataset is collected over 17 years of claims data at Fetch, Inc. Two aspects are important to consider:

- *Age at inception*: the age at which dogs were first insured
- *Latest age*: for active policies this reflects the dog’s current age. For deceased dogs and/or for dogs no longer covered by a policy this reflects their age at the end of their policy coverage period

**Figure 1:**
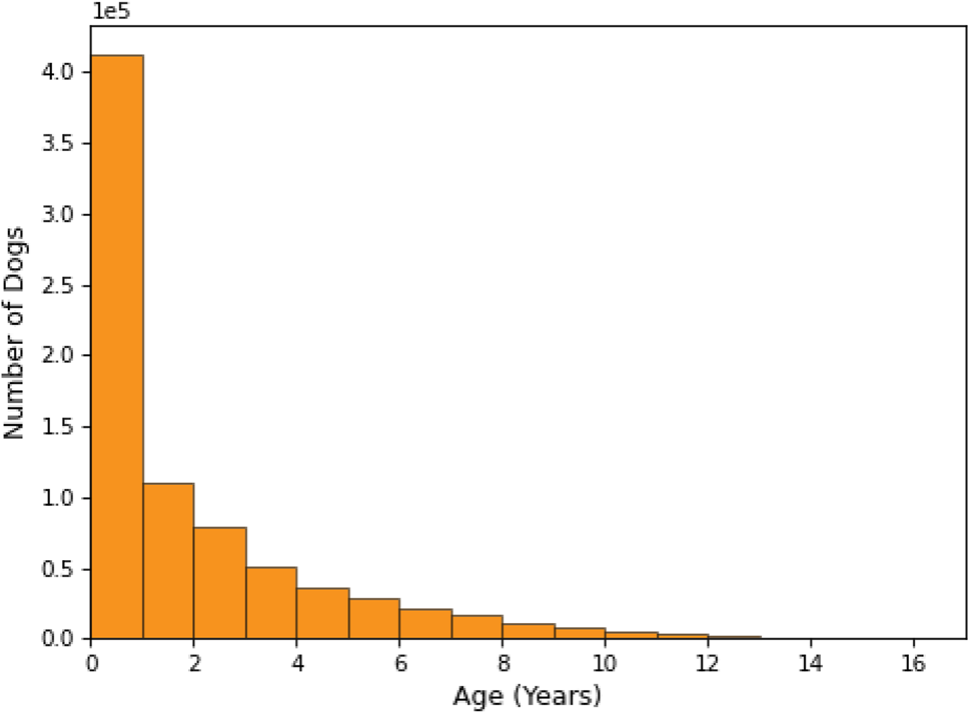
Distribution of Age at inception.

**Figure 2:**
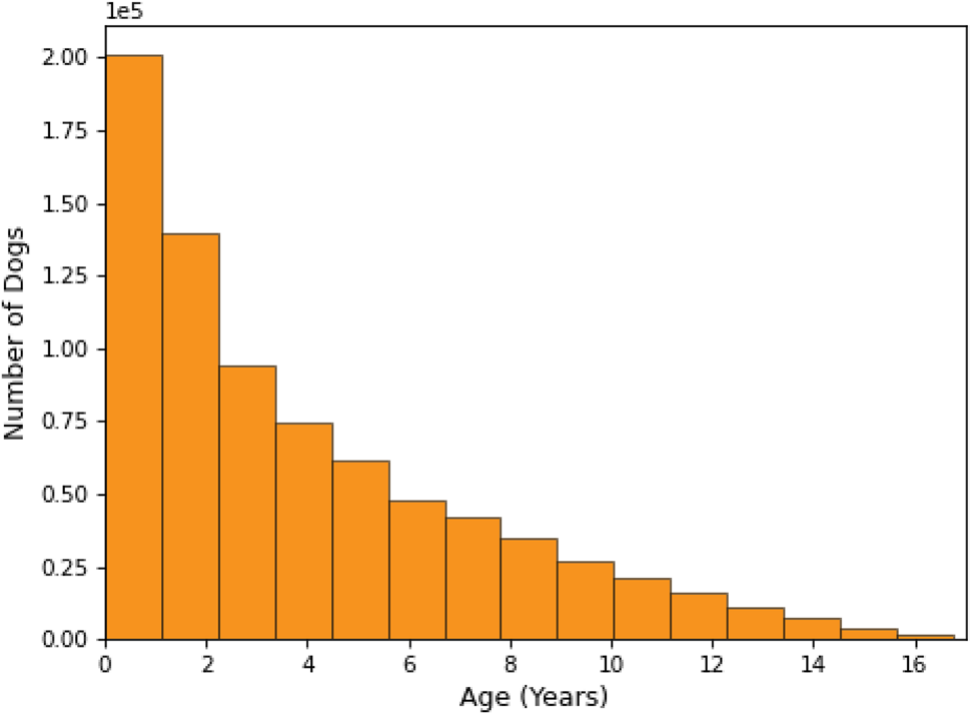
Distribution of latest age.

Figures 1 and 2 represent the distribution of each of those age groupings. We note that 52.5% of dogs were insured within the first year of their life. From a statistical and machine learning perspective, it is worth noting that dogs with a higher age at inception, bring a greater uncertainty with regards to previous disease occurrences. Thus, for all downstream analyses, we consider dogs with an age at inception $\le$ 1 year.

Also, as can be seen in Fig. 2, young dogs are over-represented in the dataset. This is mainly due to the growth of Fetch, Inc.’s business in the recent years as most dogs are a young age when they are initially insured.

#### Location

Fetch, Inc. offers insurance within the United States (US) and Canada where location information is collected via zip codes or Forward Station Areas (FSA), respectively. Whilst this information is not directly applicable to the machine learning model, it is processed in the pre-processing steps. Environmental features, such as human population density, median household income^36,37^, total annual precipitation, and average annual temperatures^38^ were mapped to zip code or FSA. Through this mapping step, the model profits from correlations between the occurrence of diseases and environmental parameters.

### Preprocessing

As mentioned in the previous section, for two of the variables (breed as one independent variable and conditions as both dependent and independent variable) we first had to ensure the availability of significant sample sizes. The machine learning algorithms that will be described in the later sections predict on a yearly basis meaning that a sample is defined as one year history of an individual dog. A dog that was insured for 10 years would thus generate 10 samples. We found empirically that 5000 disease claims represent a large enough sample size per disease group to be predicted. Disease groups that are reflected as target variables with less than 5000 claims can lead to large variations in the later cross-validation and are therefore excluded. It is expected that as the dataset increases in the coming years the list of predictable diseases will grow.

Any predictive algorithm will only work well if enough representative samples from which it can learn are present in the dataset. Similarly to how breed categories were created as described in the previous section, grouping of disease-related claims was also necessary as is described in the following.

#### Disease grouping

Disease-related claims were organized hierarchically based predominantly on the VeNom standard set of clinical veterinary terms^39^ so that similar disease outcomes that were classified using different terms could be grouped together in the analyses. For instance, the outcomes coded as intervertebral disc disease, herniated disc, ruptured intervertebral disc, and prolapsed intervertebral disc were combined into a single group called "disc diseases". Each outcome was mapped to only one disease group. As a result of this arrangement, 45 disease groups are considered for predictions in the model. These disease groups consist of 710 specific diseases and they represent 82.5% of the submitted claims (Table 3). We note that the remaining 17.5% of the submitted claims were distributed over 44 distinct disease groups. Three of these disease groups include claims related to routine treatments and surgeries which are used to improve the performance of the model but are not predicted themselves. This is further discussed in Section "Proposed approach". The remaining 41 disease groups had sample sizes too small to be considered for prediction. We refer to Supplementary Material S2 for the full mapping between claim conditions and disease groups.

**Table 3:** Frequencies of disease groups/target variables.

Disease group	Claims (%)
Vomiting and diarrhea	170,401 (6.83%)
Dermatologic immune diseases	117,586 (4.71%)
Unspecified allergies	100,458 (4.03%)
Mass lesion or swelling	100,068 (4.01%)
Ear inflammation and infections	89,762 (3.60%)
Injuries	89,215 (3.58%)
Gastroenteritis and other gi disorders	82,346 (3.30%)
Urinary tract disorders	77,965 (3.12%)
Gait abnormalities	71,323 (2.86%)
Cruciate ligament injuries	69,451 (2.78%)
Arthritis	67,123 (2.69%)
Pain disorders	64,644 (2.59%)
Mass lesion or swelling malignant	57,757 (2.31%)
Seizures	57,605 (2.31%)
Infectious disorders	54,335 (2.18%)
Respiratory infections	49,922 (2.00%)
Liver disorders	48,359 (1.94%)
Skin infections	43,699 (1.75%)
Adrenal gland disorders	41,080 (1.65%)
Oral inflammation	40,969 (1.64%)
Heart diseases	37,700 (1.51%)
Heart murmurs or arrythmias	32,720 (1.31%)
Diabetes	32,479 (1.30%)
Inflammation	31,004 (1.24%)
Eye inflammation	30,529 (1.22%)
Foreign body	28,510 (1.14%)
Leg injuries	27,626 (1.11%)
Mass lesion or swelling hematopoietic	27,614 (1.11%)
Kidney disorders	27,538 (1.10%)
Skeletal conformation disorders	27,325 (1.10%)
Thyroid disorders	24,703 (0.99%)
Internal parasites	23,601 (0.95%)
Intoxication	22,802 (0.91%)
Disc diseases	22,302 (0.89%)
Itching	19,319 (0.77%)
Dental conditions	19,111 (0.77%)
Lethargy	17,639 (0.71%)
Anxiety or phobia	17,361 (0.70%)
Eye diseases	14,620 (0.59%)
Behavioral disorders	14,344 (0.57%)
Anal gland disorders	14,035 (0.56%)
Immune disorders	13,712 (0.55%)
Urinary incontinence	12,566 (0.50%)
Digestive disorders	12,456 (0.50%)
Gastrointestinal nervous system disorders	11,907 (0.48%)

#### Breed characteristic mapping

It is well known that specific dog breeds can have predispositions to certain diseases^28,40^. This predisposition can be captured by the breed variable as a machine learning algorithm learns. However, some factors which are correlated with the occurrence of a health condition could be the same across many breeds e.g. size, coat length or behavior. It is known, for example, that large dogs share similar disease profiles, e.g. when it comes to diseases such as arthritis or cruciate ligament injuries^41,42^.

Based on this observation, we can hypothesize that some characteristic features of breeds, such as size, can help the predictive model generalize better and also improve the prediction accuracy for rare breeds. Many breed characteristics have been described by Kennel Clubs and these attributes were added to the dataset as supplemental breed-related variables^43,44^. Table 4 lists a few examples of how some characteristics (coat length, shedding, size, and trainability) vary across different breeds. Other characteristics imputed into the model include demeanor and amount of exercise required to maintain a healthy physique.

**Table 4:** Breed characteristics: examples.

Breed name	Coat length	Sheds	Size	Trainability
Affenpinscher	Easy training	Medium	Yes	Small
Afghan hound	May be stubborn	Long	Yes	Large
Bichon frise	Agreeable	Medium	No	Small
Chihuahua	Independent	Medium	Yes	Small
Golden retriever	Eager to please	Medium	Yes	Large
Great dane	Agreeable	Short	Yes	Large
Mexican hairless Dog	Agreeable	Short	No	Small
Pharaoh Hound	Independent	Short	Yes	Medium
Poodle	Eager to please	Medium	No	Medium
Siberian husky	Independent	Short	Yes	Medium

#### Environmental mapping

Besides characteristics of the breed, it is known that environmental factors can have both direct and indirect effect on the health of dogs. For instance, rates of intestinal parasitism have been shown to differ in dogs located in rural and urban environments^45^ and living in areas with extreme heat can result in heat-related illnesses in dogs^46^.

To fully leverage the knowledge of zip-codes or FSAs for each dog in the dataset, the primary data set is further enriched with the residential and climate information, such as the population density and average temperature of the corresponding area. This information is summarized in the features described in Table 5. We refer to Supplementary Material S3 for examples of residential and climate features for zip-codes or FSAs.

**Table 5:** Residential and Climate Features.

MHHI	Median household income
POPD	Population density
TAVG	Average annual temperature (in ${\circ }$C)
PRCP	Total annual precipitation
DP01	Number of days with precipitation $\ge$ 0.01 inches (0.254 mm)
DP10	Number of days with precipitation $\ge$ 1.00 inch (2.54 mm)
DT32	Number of days with minimum temperature $\le$ 32${\circ }$F (0 ${\circ }$C)
DX70	Number of days with maximum temperature $\ge$ 70${\circ }$F (21.1 ${\circ }$C)
DX90	Number of days with maximum temperature $\ge$ 90${\circ }$F (32.2 ${\circ }$C)

### Proposed approach

In this section, we present the mathematical problem formulation of disease prediction, the proposed solutions, and an evaluation framework that allows us to objectively compare different feature engineering, machine learning, and ensemble strategies.

#### Problem formulation

It is our aim to devise a system of likelihood estimation of a dog contracting a set of *M* diseases. Let $\underline{\textbf{y}}$ represent the binary vector of disease contractions over the next year with $y_m$ being its *m*-th element. $y_m \in \{0, \, 1\}$ where $y_m = 0$ denotes the absence of the *m*-th disease and $y_m = 1$ denotes its presence.

Further, let $\underline{\textbf{f}} \in \mathbb {R}^D$ denote the *D*-dimensional feature vector containing the individual, breed and environmental features as detailed out in the previous section.

The problem formulation is a multi-class classification problem, where a set of functions:

1 $$\begin{aligned} \phi _{m} \; : \; \underline{\textbf{f}} \mapsto y_{m} \end{aligned}$$

mapping the feature vector to the binary label of the $\textit{m}^{th}$ disease, needs to be established.

As we are mostly interested in disease probabilities and not binary outcomes we estimate the likelihood of the *m*-th disease label being equal to 1 as,

2 $$\begin{aligned} \textbf{P} (y_{m} \, = \, 1 \ \mid \ \underline{\textbf{f}}) \quad , \forall \, m = 0, 1, 2 \ldots M - 1 \end{aligned}$$

#### System approach

**Figure 3:**
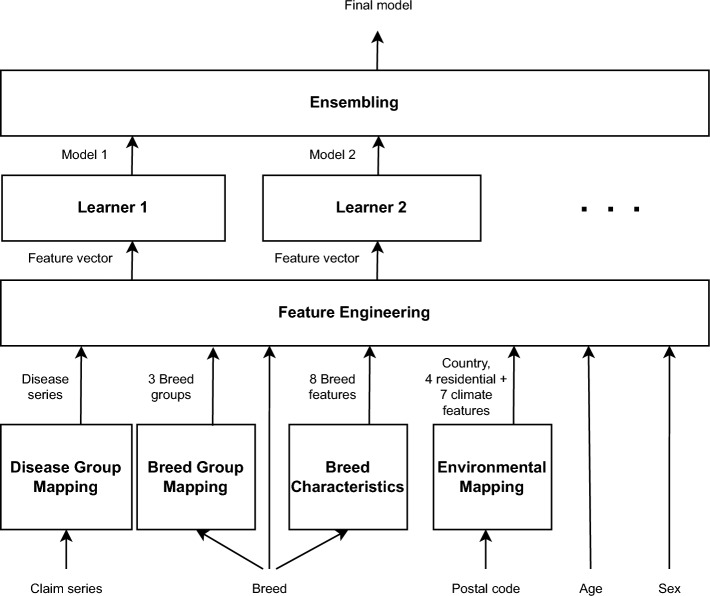
Proposed approach for disease prediction.

Our proposed approach consists of three steps as depicted in Fig. 3.

- *Feature engineering*: Based on all data points of a dog, a feature vector $\underline{\textbf{f}}$ is generated that is a numerical representation of all breed, residential, environmental and disease information of a dog.
- *Machine learning*: From the feature vector described above, a set of supervised machine learning approaches including Gradient Boosting and Logistic Regression is presented which are used for prediction.
- *Ensembling*: An ensembling framework is used to combine the advantages offered by the individual machine learning approaches.

#### Feature engineering

The health prediction system aims at estimating the disease likelihood over one year. This is realized by producing binary target variables, indicating the occurrence of a disease (more precisely, the filing of at least one claim related to said disease), over the course of a year.

The raw data is transformed/re-sampled in a way that each data sample represents the disease history of a single dog over a span of one year, while capturing the essence of the entire recorded disease history until the end of the corresponding year. This is done by creating two features for each disease group:

1. *Last*: binary value indicating whether at least one claim related to the disease was filed in the last year
2. *Average*: total number of filed claims divided by the insurance duration

In addition to the claims from the 45 disease groups which are predicted, the aforementioned sets of disease features are also produced for three disease groups (”Preventive”, ”Treatment” and ”Surgical”) covering claims related to routine treatments and surgeries. This results in a total of $(45 + 3)\cdot 2 = 96$ disease related features.

Table 6 lists the type of all other variables which are used to build the full feature vector. All categorical features are one-hot-encoded. To capture breed information three different groupings (breed group, sub breed, breed type) in addition to the most common 50 breeds are considered. Details on these are provided in supplementary material S1. Area type includes "urban" vs. "suburban" vs. "rural". "Not recorded" categories are also introduced for categorical variables with missing data e.g. for variable "sex", the categories are "Male", "Female" and when the sex information is not available; "Not recorded".

Finally, equally spaced numbers between 0 and 1 are assigned to the values of ordinal variables e.g. for Coat Length, the values are mapped as {"Short": 0, "Medium": 0.5, "Long": 1}. The number of features extracted from each variable or variable group is listed in Table 7.

**Table 6:** Types of variables.

Categorical	Breed Group, Sub Breed, Breed Type, Breed, Sheds, Trainability, Exercise, Demeanor, Country, Area type, Sex
Numerical	Age, Total population, MHI, POPD, TAVG PRCP, DT32, DP01, DP10, DX70, DX90
Ordinal	Energy level, Coat length, Size

**Table 7:** Number of features.

Variable	Feature count
Disease	96
Breed	51
Breed group	20
Sub breed	30
Breed type	10
Age	1
Environmental	10
Sex	3
Breed characteristics	22
Country	2
Area type	4
Total	249

#### Machine learning

We present 6 supervised learning methods as a solution to the classification problem posed in Section "Proposed approach". A brief summary of these methods is as follows:

- *Naive Bayes* makes a simplistic assumption on features being independent and minimizes the cost of misclassification. Despite its naive design this algorithm can sometimes outperform more complex models as it relies on much fewer samples to be available, actually avoiding the curse of dimensionality^47^ through the assumption of independent features. We note however that “Naive Bayes” is included as a very common baseline model, knowing that its assumption on independent features and potential occurrence of the zero-frequency problem will not make it an appropriate choice for this application^48^.
- *Support vector machine* determines an optimal hyperplane for segregating the classes in the feature space. This is done by maximizing the distance to the support vectors i.e. points in the features space, closest to hyper-plane. As only the support vectors are used for optimization, the risk of over-fitting is relatively small^49^.
- *Logistic regression* models the likelihood $\textbf{P} (\underline{\textbf{f}} \mid y_{m})$ as a logistic function, whose coefficients can be determined by maximum likelihood estimation^50^.
- *Multilayer perceptron* is a universal function approximator. The feature vector acts as the input to a multi-layered neural network with 2 (soft-maxed) outputs (1 for each binary label)^51^.
- *Gradient tree boosting* builds a decision tree in an iterative fashion. At each iteration, higher weights are assigned to the data samples with a higher prediction loss in the previous iteration, resulting in a tree with reduced bias but a higher variance and prone to over-fitting^52^.
- *Extreme gradient boosting* (XGBoost) builds on the same idea as gradient tree boosting being more efficient and scalable through various approximations, e.g. in approximating the loss function^53^.

#### Ensembling

Machine learning models have varying strengths and weaknesses. For example, one model may perform well for a subset of classes in a classification problem and another model might perform well for a different subset of classes. It would be natural to ask whether these two models could be combined into a model which outperforms these individual models. Ensemble learning focuses on combining the strengths of machine learning models by combining them into a stronger model^54,55^.

An ensemble can be formed from models of the same kind or from models of different kind e.g. training a logistic regression and a gradient boosting model for a classification problem and averaging the probabilities predicted by both models.

### Experimental Setup

**Figure 4:**
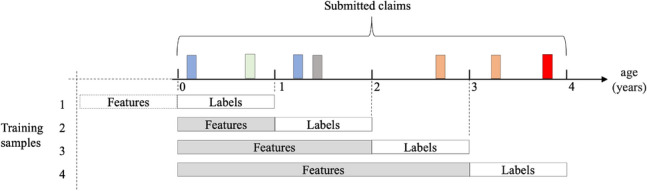
A diagram illustrating how the training samples are generated from the disease history. The colored bars represent examples of different disease claims on the age axis.

From the full dataset of 785,565 dogs, we first generated a subset of samples that can be used in the training phase of the classifier. From each year of disease history, we generate samples for target variables and use the preceding disease history to generate the corresponding features. Therefore for a dog with *n*-years of disease history, there will be *n* training samples. This concept is illustrated in Fig. 4.

We note that while most dogs in the database were initially insured within the first few weeks after birth, several were insured at a much later age. For such dogs, there is less reliable and incomplete information on their disease history, which is why we excluded dogs with an insurance policy that was initiated at the age of one year or older from the training phase. Also, as the machine learning algorithm predicts one year in the future we can only consider dogs with at least 1 year of history for the training. We note that this is only a restriction in the training phase whereas in the inference all dogs are considered. After this cleaning phase, 270,203 dogs with 1,025,099 claims remained in the final dataset.

The dataset is split using a 5-fold cross-validation with 80% of the data in training and 20% in validation^48^. During each split it is ensured that the sets are representative in terms of the distribution of breeds, age, sex and disease groups. As this is an unbalanced classification problem^56,57^, we considered the average area under curve (AUC)^58^ as a metric to summarize the classifiers performance when it comes to predicting all 45 disease groups. The AUC is defined as the area under the "probability of detection" versus "probability of false alarm" curve. An AUC of 50% represents the so called chance line, i.e. a classifier that performs as good as flipping a coin. The AUC can reach a theoretical value of 100% which would correspond to a scenario in which the classifier has 100% probability of detection at 0% false alarms. A set of experiments was conducted which are described in the following.

#### Experiments 1: richness of features

The first set of experiments is aimed at understanding the richness of input factors. The idea is to understand the joint and partial contribution of each of the factors such as disease history, breed or the environment a dog is living in, to the disease prediction. We consider two experiments as the (naive) baseline experiments:

- *EXP 1-1*, in which the machine learning model only has information on the disease history, thus constructing a model for the average dog that ignores the fact that many diseases are more prominent for certain breeds, for male or female dogs or in warmer or more populated regions.
- *EXP 1-2*, in which the machine learning model only has information on the breed and age, thus constructing an average dog per breed, ignoring important factors such as an individual disease history.

The following Table 8 summarizes the resulting 11 experiments that are considered as part of this experiment set and that have an increased richness and complexity of features.

**Table 8:** Experiment set 1 to evaluate the feature relevance.

Experiment	Diseases	Individual breed	Breed group	Age	Sex	Residential	Climate	Breed characteristics
EXP 1-1	X							
EXP 1-2		X						
EXP 1-3		X		X				
EXP 1-4	X	X						
EXP 1-5	X	X	X					
EXP 1-6	X	X	X	X				X
EXP 1-7	X	X	X	X	X			
EXP 1-8	X	X	X	X	X	X		
EXP 1-9	X	X	X	X	X		X	
EXP 1-10	X	X	X	X		X	X	
EXP 1-11	X	X	X	X	X	X	X	X

We note that for this set of experiments the actual machine learning method is kept constant and gradient tree boosting was chosen here exemplary—the results are qualitatively however similar for all other classifiers.

#### Experiments 2: model choice

EXP 1-11, i.e. the model with the richest set of features is considered as a baseline for this set of experiments. Fixing the feature set we aimed at understanding the performance of a variety of machine learning algorithms. The specific algorithms tested are in Table 9.

**Table 9:** Experiment set 2 to evaluate different machine learning algorithms.

Experiment	Algorithm
EXP 2-1	Naive Bayes
EXP 2-2	Support vector machine
EXP 2-3	Gradient tree boosting
EXP 2-4	Logistic regression
EXP 2-5	Multi layer perceptron
EXP 2-6	Extreme gradient boosting

#### Experiments 3: ensembling

The final set consists of a single experiment 3-1 designed to understand the impact of ensembling on top of the best two performing classifiers from experiment group 2. The final classifier is obtained through averaging the class probabilities of the individual classifiers.

## Results and discussion

### Results and discussion

In this section we present the results from the experiments described in Section "Experimental Setup". Section "Feature richness" focuses on the richness of features provided to the model, while Section "Machine learning models" and "Ensembling" discusses the pros and cons of different machine learning algorithms. A deep dive into feature importances and discussion of the predictive power for individual diseases is then provided in Section "Discussion".

#### Feature richness

The following Table 10 shows the training and crossvalidation AUC’s for the 11 experiments described in Section "Experiments 1: richness of features". The reported numbers show the average AUC over all 45 disease classes. As expected, the lowest performance is achieved by EXP 1-2 which as its only feature has the individual dog breed. We still note, that even this model has predictive power with an AUC well above 50% as it is able to model breed predispositions. Examples include increased risk for dermatological immune diseases for English bulldogs, increased risk for disc diseases for dachshunds and increased risk for skeletal conformation disorders for German shepherds.

The second ’naive’ model, EXP 1-1, which is unaware of breed and age, but captures the individual disease history, already performs significantly higher, reaching a test AUC of 75%. This clearly shows that on its own, breed information is somewhat useful for disease prediction, but it is much more informative when combined with the disease history. Examples include increased risk for various dental conditions after diagnosis of oral inflammation, increased risk for behavioral disorders after diagnosis of anxiety and phobia, and increased risk for arthritis after cruciate ligament (knee) injuries.

We observe the power of combining the individual disease history with breed information in EXP 1-4 which gives an increase of 10-15 percent in overall AUC compared to the breed-only model EXP 1-2 and the disease-only model EXP 1-1.

EXP 1-5 which adds the breed group as an additional feature further increases the performance . We note that this feature is less relevant for breeds with a high population such as golden retrievers or German shepherds, but it strongly increases performance for less numerous breeds that by themselves do not have enough samples to build a strong independent model but can profit from similar breeds that are in the same breed group.

For all further experiments 1-6 to 1-11 we observe a slight increase in performance with the full-feature model EXP 1-11 having the highest performance. It is notable that the addition of breed characteristics in EXP 1-11 adds a small performance boost due to the same reason as for the breed grouping feature; breeds with small population sizes can profit from training samples from dogs of different breeds that share similar characteristics such as size, coat length, etc.

**Table 10:** Area under the ROC-Curve for Experiment Set 1.

	Train AUC	Crossval AUC
EXP 1-1	75.24%	68.69 ± 1.85%
EXP 1-2	61.43%	60.43 ± 0.82%
EXP 1-3	75.64%	67.81 ± 0.81%
EXP 1-4	77.79%	71.09 ± 1.44%
EXP 1-5	78.61%	71.10 ± 1.31%
EXP 1-6	81.75%	73.75 ± 1.43%
EXP 1-7	81.68%	73.58 ± 1.69%
EXP 1-8	82.24%	74.66 ± 1.67%
EXP 1-9	83.04%	74.85 ± 1.46%
EXP 1-10	84.08%	75.02 ± 1.49%
EXP 1-11	84.14%	75.51 ± 1.58%

#### Machine learning models

Table 11 shows both the training as well as cross-validation AUCs obtained when testing all six machine learning approaches on the full-feature dataset. As in the previous section, the AUCs reported show the mean of the 45 AUCs for all 45 predicted diseases. We can observe that Naive Bayes shows the lowest performance, clearly a sign of the independence assumption of features since for example disease history and age are naturally dependent.

All other models that are able to capture the dependence of features perform significantly higher with extreme gradient boosting showing the highest overall AUC. We further observe that the multilayer perceptron has a tendency to overfitting with a cross-validation AUC that is almost 20% below the training AUC.

**Table 11:** Area under the ROC-Curve for Experiment Set 2.

		Train AUC	Crossval AUC
Naive Bayes	EXP 2-1	73.77%	72.62 ± 0.92%
Support vector machine	EXP 2-2	77.59%	77.51 ± 0.77%
Gradient rree boosting	EXP 2-3	84.14%	75.51 ± 1.58%
Logistic regression	EXP 2-4	79.55%	77.97 ± 0.79%
Multilayer perceptron	EXP 2-5	92.90%	73.64 ± 1.11%
Extreme gradient boosting	EXP 2-6	90.34%	80.60 ± 0.72%

**Figure 5:**
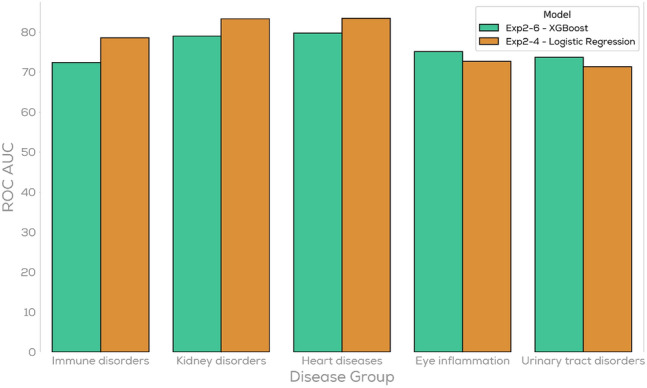
Comparison of the test AUC scores for Extreme Gradient Boosting and Logistic Regression on different disease categories.

When investigating the individual AUCs for all 45 disease categories we observe that different algorithms perform better for different diseases. This difference is visually shown in Fig. 5 where the cross-validation of five selected diseases (Immune disorders, Kidney disorders, Heart diseases, Eye inflammation, and Urinal tract disorders) are shown for both the Extreme Gradient Boosting as well as Logistic Regression algorithm. While for the first three diseases logistic regression outperforms Extreme Gradient Boosting we observe the opposite for the latter two diseases. Generally it seems that Logistic Regression has advantages on classes with lower sample sizes while Extreme Gradient Boosting performs better for classes with high sample sizes.

These results clearly show the benefit of using ensembling techniques such as combining multiple models as will be discussed in the following section.

#### Ensembling

In order to benefit from ensembling it is of importance to combine models that show good performance individually, but also have good performance in different settings. This is achieved as demonstrated in the previous section for example for Logistic Regression and Extreme Gradient Boosting that both have a high individual performance (significantly above the chance line of 50%) while having individual strengths and weaknesses.

The following Table 12 shows the experimental result of combining these two algorithms. Ensembling here is achieved through soft voting. Mean cross validation scores for each disease are used to calculate the weights of the models for that disease and the weighted sum of the predicted probabilities is used as the final probability of the occurrence of the disease. An increase in the cross-validation AUC of 0.6% is achieved as compared to Extreme Gradient Boosting alone.

**Table 12:** Area under the ROC-Curve for Experiment Set 3.

		Train AUC	Crossval AUC
Ensemble of XGBoost and linear regression	EXP 3-1	88.56%	81.23 ± 0.73%

**Figure 6:**
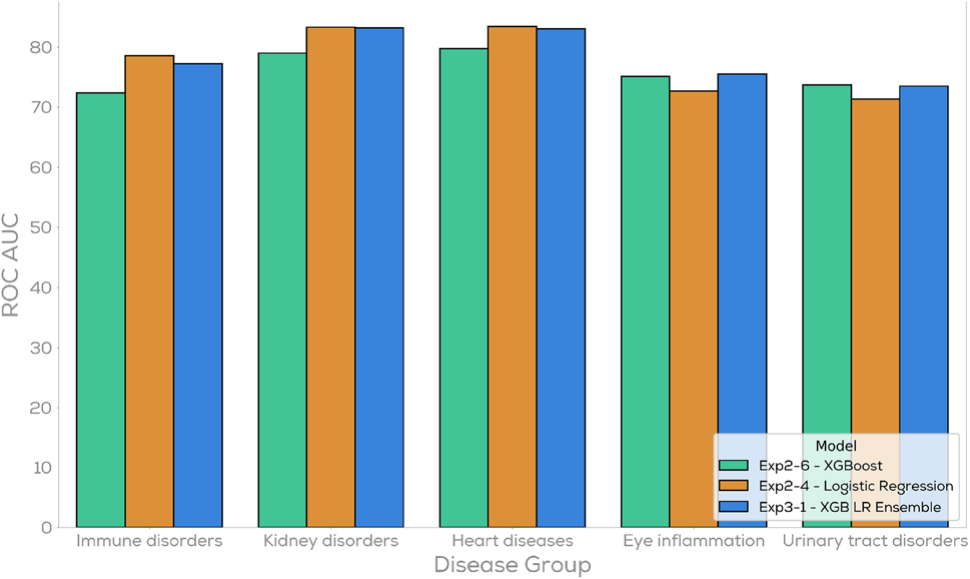
Comparison of the test AUC scores for Extreme Gradient Boosting, Logistic Regression and Ensemble on different diseases.

When comparing the individual AUC scores of the five diseases shown in the previous section we observe, as per Fig. 6 that the ensemble model tends to converge to the higher score of the individual models.

#### Discussion

This work shows that in terms of overall training and crossvalidation, it is of importance to examine individual diseases or disease groupings and understand both their predictability as well as the respective feature importances. Regarding the individual predictability of diseases we determined the AUCs for all 45 disease groups, both in training as well as on a 10% test dataset (Table 13). Our experiments also show that some disease categories have a very high predictability. These include arthritis, disc diseases, diabetes, hematopoietic cancers, thyroid disorders, and adrenal gland disorders. These are conditions that are either chronic (i.e. the model learned that the presence of previous claims is a strong indicator for future claims of the same kind) or for which certain constellations of age and breed allow for high prediction performance.

Other disease categories such as vomiting and diarrhea, foreign body ingestion and soft tissue injuries all have significantly lower AUCs as they are inherently more difficult to predict. There are certain breeds and age groups for which these conditions happen more often, which is reflected in AUCs that are significantly above the 50% chance line but overall these types of incidents would naturally occur in a less predictable fashion. We also note that for some conditions, such as dental conditions or immune disorders, we still observe a larger discrepancy between train and test AUC which might indicate a certain degree of overfitting.

**Table 13:** Area under the ROC-Curve for individual disease groups (XGBoost).

Disease group	Train AUC (%)	Crossval AUC (%)
Diabetes	99.90	94.66 ± 1.28
Mass lesion or swelling hematopoietic	99.63	88.97 ± 0.91
Adrenal gland disorders	98.99	90.68 ± 0.88
Eye diseases	98.92	89.59 ± 0.97
Immune disorders	98.00	80.66 ± 2.15
Thyroid disorders	97.96	90.86 ± 0.09
Disc diseases	97.63	88.41 ± 0.50
Kidney disorders	96.85	81.07 ± 2.15
Mass lesion or swelling malignant	96.51	89.49 ± 0.47
Arthritis	96.36	89.94 ± 0.51
Urinary incontinence	96.31	85.53 ± 1.03
Heart diseases	95.89	83.29 ± 1.45
Skeletal conformation disorders	95.70	89.00 ± 0.76
Heart murmurs or arrythmias	95.36	85.25 ± 0.56
Behavioral disorders	94.67	83.61 ± 0.91
Liver disorders	94.28	85.10 ± 1.11
Digestive disorders	93.69	76.93 ± 0.90
Seizures	92.79	80.30 ± 1.07
Anxiety or phobia	92.45	81.11 ± 0.69
Internal parasites	92.37	82.28 ± 0.76
Gastrointestinal nervous system disorders	91.87	72.16 ± 0.80
Oral inflammation	91.63	86.75 ± 0.39
Anal gland disorders	91.45	77.88 ± 0.95
Itching	90.54	79.69 ± 0.65
Cruciate ligament injuries	90.50	84.47 ± 0.10
Unspecified allergies	89.87	84.97 ± 0.33
Lethargy	88.30	69.30 ± 1.00
Inflammation	87.81	75.07 ± 0.86
Dermatologic immune diseases	87.15	83.15 ± 0.34
Eye inflammation	87.00	79.16 ± 0.42
Dental conditions	86.59	72.53 ± 0.98
Skin infections	85.89	77.89 ± 0.75
Pain disorders	85.72	75.73 ± 0.44
Leg injuries	84.97	73.98 ± 0.69
Respiratory infections	84.65	74.02 ± 0.59
Urinary tract disorders	84.31	76.91 ± 0.35
Mass lesion or swelling	83.88	78.66 ± 0.40
Foreign body	83.83	74.62 ± 0.42
Gastroenteritis and other gi disorders	83.32	75.48 ± 0.40
Intoxication	83.13	71.04 ± 0.43
Ear inflammation and infections	82.19	77.51 ± 0.33
Gait abnormalities	81.92	73.81 ± 0.57
Infectious disorders	81.39	73.47 ± 0.52
Injuries	76.84	69.75 ± 0.51
Vomiting and diarrhea	76.18	72.06 ± 0.12
Mean	90.34	80.60 ± 0.10

Clearly, on a topic such as health prediction, one would aim for explainable AI^59,60^, as in models that can not only provide predictions, but also explain in an understandable way how it came to those predictions, such as a high risk for arthritis or malignant tumors. This is extremely important for use in veterinary research in order to understand which combinations of breed, previous disease history, and environmental factors yield increased risks for which disease outcomes. It is also highly important for use in creating recommendations for individual dogs as the understanding of which features are driving the increased risk can have an immediate impact on the preventive methods that can be employed to reduce the risk and avoid the disease occurrence.

**Figure 7:**
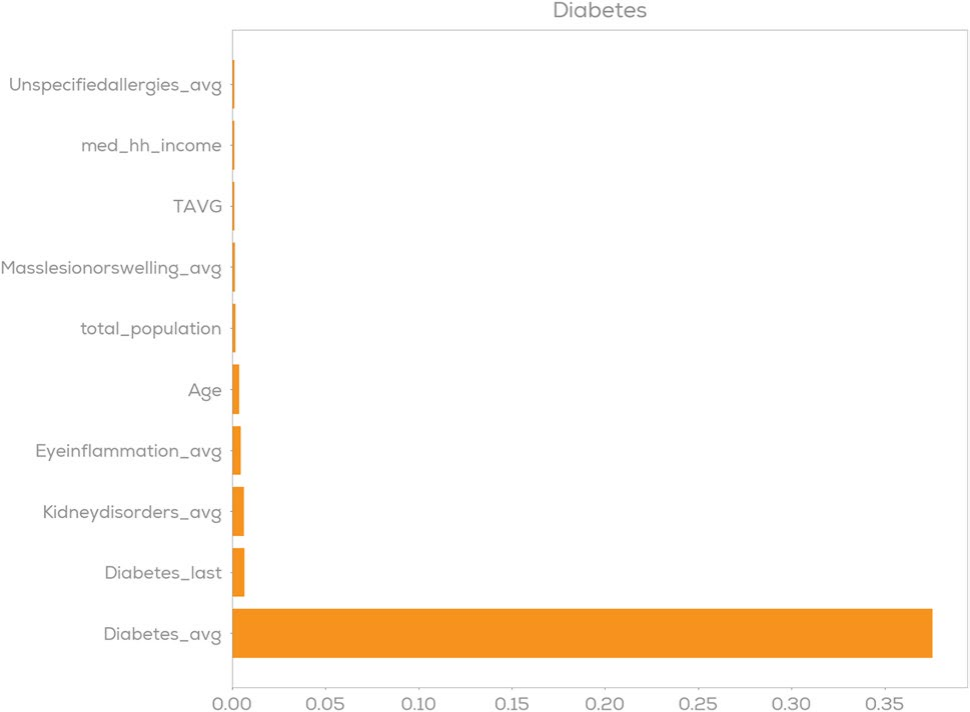
Feature importance plot for diabetes prediction.

**Figure 8:**
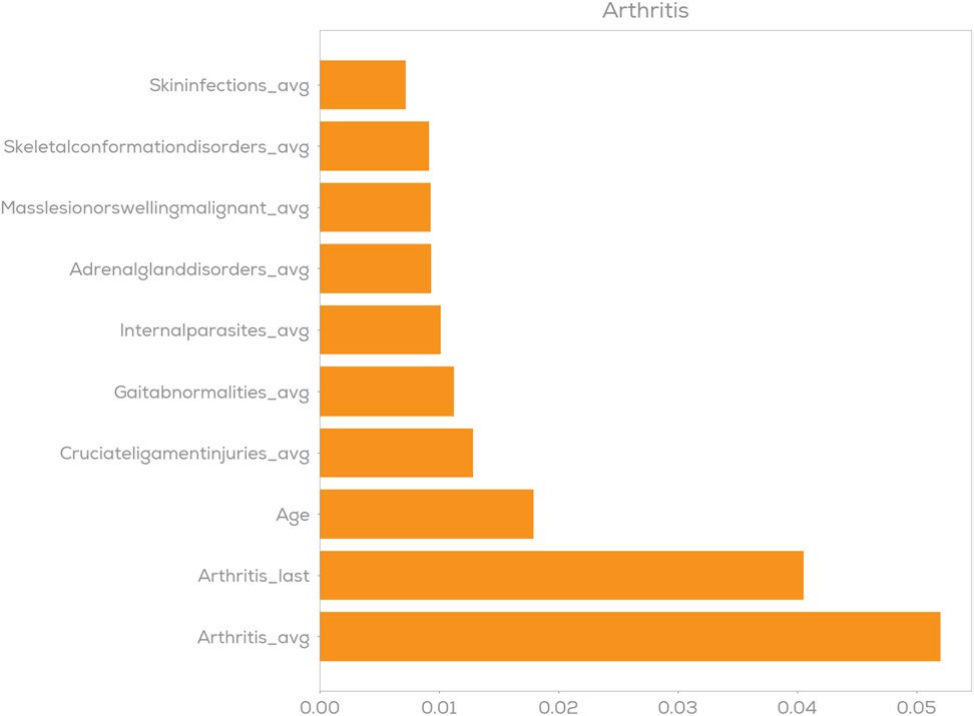
Feature importance plot for arthritis prediction.

As a first indicator for model explainability, we consider feature importance plots^48^. For an individual disease category these indicate the relative importance of individual features such as age, breed or previous diseases. In Figs. 7 and 8 feature importance plots for diabetes and arthritis are shown using the XGBoost model as an example. The suffix _last and _avg in these plots represent the disease-related features that are described in Section "Proposed approach"—_last representing a binary value on whether a respective claim was filed in the last year and _avg representing the average number of filed claims per year. One can observe that for diabetes prediction the model learned that the strongest predictor is the presence of previous diabetes claims and only very minor contributions come from e.g. previous claims on kidney disorders or age. For arthritis prediction as shown in Fig. 8 we observe a different scenario: Still, the disease category itself (here: represented via Arthritis_avg and Arthritis_last) are the two strongest indicators, but we have a much larger group of features that are indicative for Arthritis prediction. These naturally include age, but also the presence of cruciate ligament injuries or gait abnormalities. These are confirmed by existing veterinary research^42,61^.

**Figure 9:**
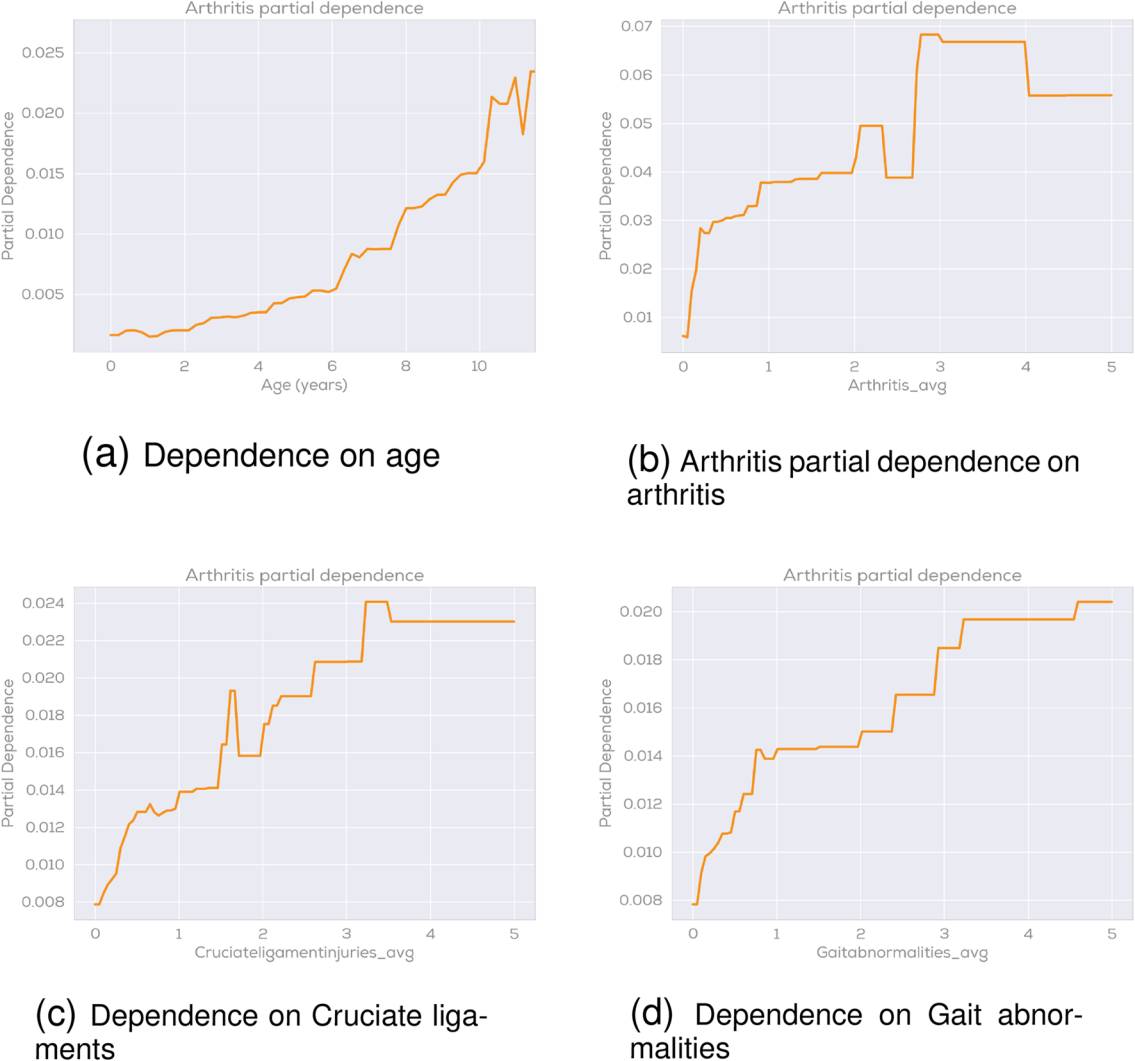
Partial dependence plots for arthritis prediction.

Feature importance plots are only a first indicator and don’t show the direction of the importance. As an example, the feature importance plot for arthritis shows that the model learned that age is an important feature with high predictive power but it doesn’t show whether e.g. a higher or lower age correlates with an increase in arthritis claims. As a next level indicator we used partial dependence plots (PDPs)^62^ in order to analyze predictions. PDPs show the dependence between the target response (in our case the presence of a certain disease) and an input feature (e.g. age). The following (Fig. 9) shows an example of such PDPs for the case of arthritis prediction. In Fig. 9a the dependence of age on arthritis prediction is shown. As expected we see the PDP plot being close to zero in the puppy age and constantly increasing with age, reaching its maximum in the oldest age category. Figure 9b shows the dependence of the average number of previous arthritis diagnoses per year on the prediction of future claims related to arthritis diagnoses. This ”self-prediction,” where once a disease has occurred there is a higher likelihood of claims related to it in the future, is observable over all disease group categories and is typically one of the strongest predictive features. In the PDP plot we observe an increasing trend meaning a high number of previous arthritis claims is indicative for having more arthritis claims.

Figure 9c and d then show the dependence of the average number of cruciate ligaments and gait abnormalities claims per year on arthritis prediction. Both plots show that these previous conditions have a clear impact on arthritis prediction.

#### Limitations

In addition to the limitations included in the discussion above, it is important to remember that the outcome data used for predictive modeling were compiled exclusively by an insurance provider and thus are considered a secondary data source^63^. Though some data was robustly reported for all individual dogs included in the dataset, like address information, other information about the total population may have been reported with lesser accuracy. For instance, some locations in the United States enforce breed-restrictions and thus dog owners may have inaccurately reported their dog’s breed to the insurance provider.

Another possible limitation is the use of breed groups to overcome small sample sizes in the dataset. This could result in breeds with higher population numbers within the dataset influencing the results of predictions of breeds that are less well represented. Further, the inclusion of breed characteristics developed by Kennel Club organizations may be oversimplified as there is no indication as to the degree to which specialists would agree on the categorizations (e.g. how trainable a particular breed of dog may be) attributed monolithically to each breed.

## Conclusion

In this paper we demonstrated how machine learning models can be trained on insurance claims data to predict pet health. Several machine learning models were trained to predict 45 disease group categories and evaluated in terms of their predictive power. Models with rich features ranging from breed characteristics, individual disease history, sex, as well as environmental features derived from the dogs geographic location showed the highest performance. It was also shown that the combination of machine learning models in an ensemble learning framework improves the model prediction accuracy even further.

Besides the raw AUC numbers, we demonstrated that the concept of explainable AI, specifically the use of feature importances and partial dependence plots, help to understand how the predictive model came to certain conclusions about increased or decreased risks for specific diseases. The developed machine learning models have a high enough predictive power to be used for a multitude of applications. These include applications for pet insurance companies, including building adaptive recommendation generating systems which can provide dog owners with specific preventive health recommendations with the aim of reducing disease occurrences which benefits the health of the animal and the financial outlay for the insurance provider. Other applications may include creating health reports for insured dogs, applications for veterinarians to raise awareness of individual dog risks in their clinical setting and a strong supporting dataset for research studies in veterinary medicine.

Future work on this topic will include the addition of new features, specifically additional transformations of the claim time-series as well as prescription-based features (e.g. vaccination and flea and tick treatment usage). Work on the model itself will include neural networks beyond the multilayer perceptron and parallelizable models to cope with the expected increase in sample size. Also, work in the area of ensemble learning will follow. The results reported here are promising and we expect that more potential will be found in combining additional models and applying different combination strategies. Finally, as shown in the experimental results section, we still observe a certain discrepancy between performances on the train vs test sets for individual diseases which will be addressed via more sophisticated ensembling approaches.

## Supplementary Information

Supplementary Information.

Supplementary Information.

Supplementary Information.

## Data Availability

The data that support the findings of this study are available from Fetch, Inc. but restrictions apply to the availability of these data, which were used under license for the current study, and so are not publicly available. Data are however available from Christian Debes upon reasonable request and with permission of Fetch, Inc.
